# Efficient induction and sustenance of pluripotent stem cells from bovine somatic cells

**DOI:** 10.1242/bio.058756

**Published:** 2021-11-01

**Authors:** Viju Vijayan Pillai, Prasanthi P. Koganti, Tiffany G. Kei, Shailesh Gurung, W. Ronald Butler, Vimal Selvaraj

**Affiliations:** Department of Animal Science, College of Agriculture and Life Sciences, Cornell University, Ithaca, NY 14853, USA

**Keywords:** Pluripotency, Stem cell, Embryo, Reprogramming, Culture, Differentiation

## Abstract

Although derivation of naïve bovine embryonic stem cells is unachieved, the possibility for generation of bovine induced pluripotent stem cells (biPSCs) has been generally reported. However, attempts to sustain biPSCs by promoting self-renewal have not been successful. Methods established for maintaining murine and human induced pluripotent stem cells (iPSCs) do not support self-renewal of iPSCs for any bovid species. In this study, we examined methods to enhance complete reprogramming and concurrently investigated signaling relevant to pluripotency of the bovine blastocyst inner cell mass (ICM). First, we identified that forced expression of SV40 large T antigen together with the reprogramming genes (*OCT4*, *SOX2*, *KLF4* and *MYC*) substantially enhanced the reprogramming efficacy of bovine fibroblasts to biPSCs. Second, we uncovered that TGFβ signaling is actively perturbed in the ICM. Inhibition of ALK4/5/7 to block TGFβ/activin/nodal signaling together with GSK3β and MEK1/2 supported robust *in vitro* self-renewal of naïve biPSCs with unvarying colony morphology, steady expansion, expected pluripotency gene expression and committed differentiation plasticity. Core similarities between biPSCs and stem cells of the 16-cell-stage bovine embryo indicated a stable ground state of pluripotency; this allowed us to reliably gain predictive understanding of signaling in bovine pluripotency using systems biology approaches. Beyond defining a high-fidelity platform for advancing biPSC-based biotechnologies that have not been previously practicable, these findings also represent a significant step towards understanding corollaries and divergent aspects of bovine pluripotency.

This article has an associated First Person interview with the joint first authors of the paper.

## Abstract (precis)

**Summary:** Pluripotency reprogramming by overcoming the stable epigenome of bovine cells, and uncovering precise early embryo self-renewal mechanisms enables sustenance and expansion of authentic induced pluripotent stem cells *in vitro*.

## INTRODUCTION

Leading up to the 1980s, studies on ruminants were at the forefront of developments in reproductive physiology and assisted reproduction for mammalian species (Britt et al., 1981; Willett, 1956). Derivation of murine embryonic stem cells (ESCs) in 1981 (Evans and Kaufman, 1981; Martin, 1981) opened up new possibilities for advancement, and there was significant investment in deriving bovine ESCs (bESCs). Despite preliminary successes in isolating pluripotent cells from the bovine inner cell mass (ICM) (Cherny et al., 1994; Saito et al., 1992; Sims and First, 1994; Stice et al., 1996), it was not possible to effectively sustain these cells in culture (First et al., 1994; Talbot and Blomberg, 2008). After human ESCs (hESCs) were derived in 1998 (Thomson et al., 1998), it brought to light understanding that signaling mechanisms for sustaining ESCs in culture were quite divergent. Growth factor leukemia inhibitory factor (LIF), acting via STAT3 was found to support self-renewal in mESCs (Niwa et al., 1998; Williams et al., 1988) and fibroblast growth factor 2 (FGF2), acting via activin/nodal, were identified for hESCs (James et al., 2005; Vallier et al., 2005). Numerous attempts to derive and study bESCs have continued over the years without definitive methods to sustain pluripotency and self-renewal long-term (Cao et al., 2009; Cibelli et al., 1998; Cong et al., 2014; Iwasaki et al., 2000; Kim et al., 2012; Lim et al., 2011; Mitalipova et al., 2001; Pant and Keefer, 2009; Pashaiasl et al., 2010, 2013; Saito et al., 1992, 2003; Sims and First, 1994; Stice et al., 1996; Talbot et al., 1995; Van Stekelenburg-Hamers et al., 1995; Wang et al., 2005), the most recent studies being demonstrations that a primed form of bESCs (Bogliotti et al., 2018) and other early transitional states (Huang et al., 2014) that express pluripotency markers and retain the ability to differentiate into the three germ layers can be cultured from bovine blastocysts. Hitherto, these studies have not only highlighted serious challenges to sustenance of pluripotency, but also the paucity in understanding of signaling and regulation for self-renewal in bESCs (Ezashi et al., 2016; Keefer et al., 2007; Malaver-Ortega et al., 2012).

In 2006, the discovery of induced pluripotent stem cells (iPSCs) that had characteristics identical to ESCs (Takahashi and Yamanaka, 2006; Takahashi et al., 2007), infused new enthusiasm for bovine pluripotency research. In the ensuing decade, we and others have made numerous attempts to generate bovine iPSCs (biPSCs) incorporating the four core reprogramming genes *POU5F1* (*OCT4*), *SOX2*, *KLF4* and *MYC* (*OSKM*), and other factors (Bai et al., 2016; Canizo et al., 2018; Cao et al., 2012; Cravero et al., 2015; Han et al., 2011; Heo et al., 2015; Huang et al., 2011; Kawaguchi et al., 2015; Lin et al., 2014; Pillai et al., 2019b; Sumer et al., 2011; Talluri et al., 2015; Wang et al., 2013; Zhao et al., 2017), but with limited success. Although several of these studies claim successful derivation of these cells, measures of quality have remained quite arbitrary (Pieri et al., 2019). In genome-integrating transgene based-approaches, the exogenous transgenes were not silenced (Cao et al., 2012; Cravero et al., 2015; Han et al., 2011; Heo et al., 2015; Huang et al., 2011; Sumer et al., 2011; Talluri et al., 2015; Zhao et al., 2017), or this was not evaluated (Canizo et al., 2018; Lin et al., 2014; Wang et al., 2013). In some studies, forced expression of the reprogramming genes induced trophoblast formation from bovine fibroblasts rather than pluripotent cells (Kawaguchi et al., 2016; Talbot et al., 2017). In the case of doxycycline-inducible reprogramming transgenes, continuous induction of exogenous expression was necessary to maintain biPSCs (Kawaguchi et al., 2015). Supporting the lack in activation of the endogenous pluripotency network, some studies have concluded that bovine fibroblasts present an epigenetic block that prevents complete reprogramming (Canizo et al., 2018; Kawaguchi et al., 2015). In agreement, an extrapolation that progenitors can reprogram more readily than terminally differentiated cells was confirmed (Bai et al., 2016; Kawaguchi et al., 2015; Lin et al., 2014; Pillai et al., 2019b; Wang et al., 2013). This insufficiency led to additional testing for including Nanog (Pillai et al., 2019b; Sumer et al., 2011), knockdown of p53 (Pillai et al., 2019b), knockdown of Mbd3 (Pillai et al., 2019b) and overexpression of the microRNA 302/367 cluster (Bai et al., 2016; Pillai et al., 2019b), without success. For sustenance, these studies have attempted using LIF (Heo et al., 2015; Huang et al., 2011; Lin et al., 2014; Wang et al., 2013), FGF2 (Bai et al., 2016; Canizo et al., 2018), both (Cao et al., 2012; Cravero et al., 2015; Han et al., 2011; Kawaguchi et al., 2015; Pillai et al., 2019b; Sumer et al., 2011; Talluri et al., 2015; Zhao et al., 2017) or with added BMP4 (Zhao et al., 2017), without a clear functional consensus. Together with LIF, some studies have also tried pharmacological inhibition of ﻿mitogen-activated protein kinase kinase (MAP2K1/2 or MEK1/2) and glycogen synthase kinase 3β (GSK3β) to prevent differentiation and promote self-renewal (Heo et al., 2015; Huang et al., 2011). Despite these sustained efforts, a consistent and reproducible method for maintaining a pluripotent state remains to be deciphered for cattle or for any other ruminant species (Pieri et al., 2019; Su et al., 2020).

In investigating a variety of approaches to enhance efficiency in biPSC generation (Pillai et al., 2019b), we have identified two compounding facets to the problem: (1) a stable epigenome in bovids resists iPSC reprogramming, exemplified by failures with methods that enhance permissiveness in murine and human somatic cells (Pillai et al., 2019b); (2) there is dearth in understanding of signaling necessary for sustaining pluripotency and/or differentiation specific to bovids (Ezashi et al., 2016; Pillai et al., 2019b).

To address these problems, we first experimented an approach using the simian vacuolating virus 40 large T antigen (LT), which would likely overcome any epigenetic barriers to iPSC generation (Tan et al., 2017), and then used stage-specific transcriptomics datasets to delineate pluripotency [subtracting undifferentiated trophoblast stem cells (TSCs) (Pillai et al., 2019a) from blastocyst embryos] to deduce intracellular signaling that could be preventing/suppressing post-induction long-term sustenance of biPSCs in culture. These trials culminated in a precise reproducible approach for induction and culture conditions for sustained self-renewal of naïve biPSCs. Transcriptome analysis of these biPSC in comparison to the 16-cell-stage bovine embryos (16-CEs) revealed, for the first time, underlying constants (transcriptional networks and signaling pathways) that support the bovine pluripotent cell phenotype.

## RESULTS

### Inclusion of LT considerably enhances the efficiency of inducing biPSCs

**Fig. 1.**
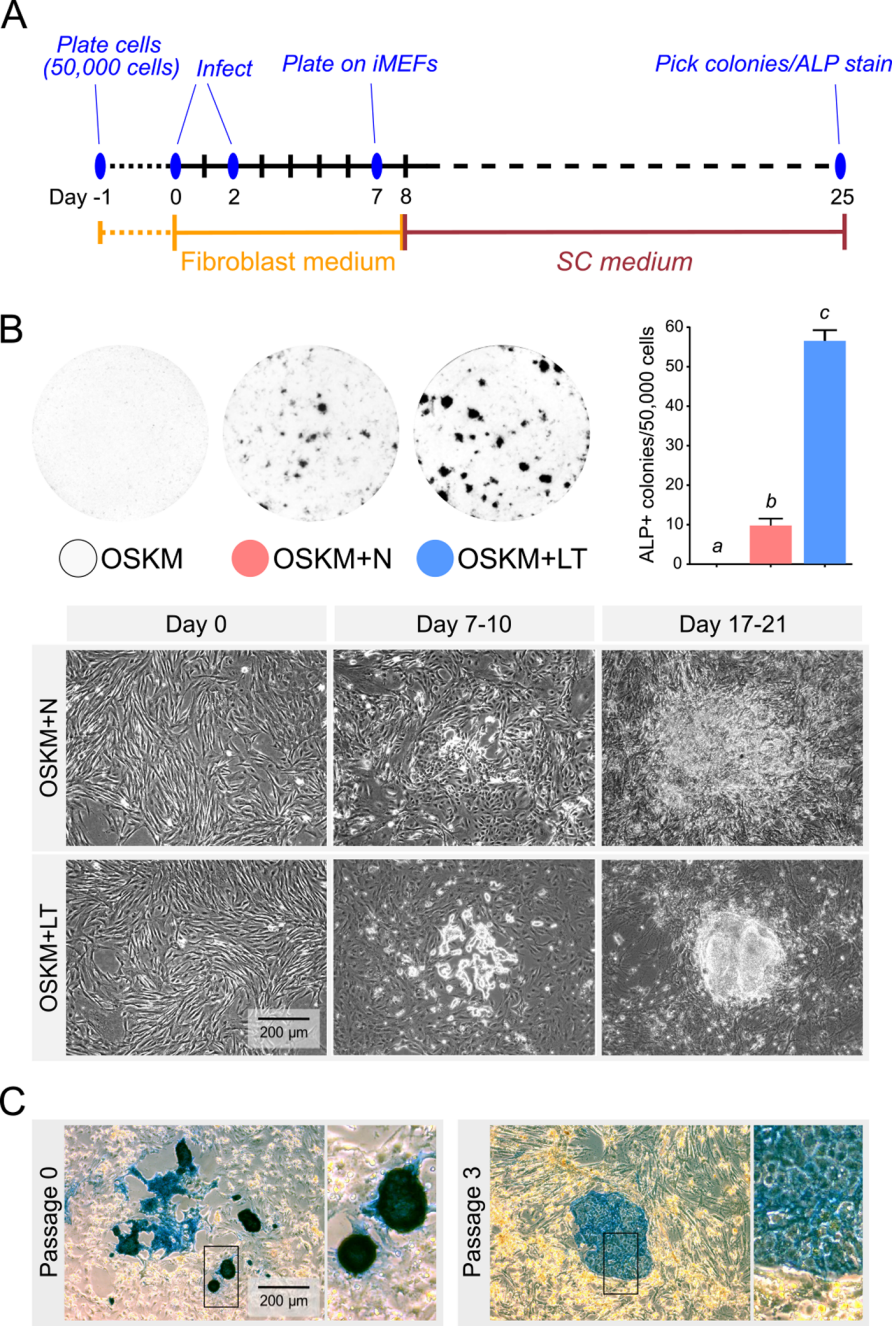
**Use of OSKM+LT dramatically enhances the efficiency of inducing biPSCs.** (A) Reprogramming method timeline showing procedures and culture conditions. (B) Top left: representative images of alkaline phosphatase (ALP)-stained plates from reprogramming experiments at Day 25 showing cultures of OSKM, OSKM+N and OSKM+LT conditions. No colonies were recorded for the condition with just OSKM. Top right: quantitative analysis confirmed a significant increase in reprogramming efficiency with OSKM-LT (*n*=5; groups not connected by the same letter are significantly different, *P*<0.05). Bottom: morphological examination of changes during the reprogramming timeline distinctively showed that OSKM+LT resulted in numerous compact colonies that contain rounded edges with individual cells not discernible. This is in contrast to the dense accumulation without rounded edges observed for OSKM+N. (C) Representative images showing ALP staining before and after passaging OSKM+LT-derived colonies in SC medium. Even after the first passage, colony morphology shifted to flattened colonies with demarcated cells and decrease in intensity of ALP staining. Colonies could not be maintained beyond the third passage.

Use of LT in addition to OSKM reprogramming factors resulted in a dramatic increase in biPSC colony formation (Fig. 1B). These colonies showed rounded edges with individual cells not being discernible. Use of OSKM alone did not result in biPSC induction, and addition of Nanog to OSKM resulted in partially reprogrammed biPSC-like cells that formed dense accumulations without rounded edges (Fig. 1B), also previously demonstrated (Pillai et al., 2019b). The compact colonies induced by OSKM+LT showed intense staining for alkaline phosphatase (ALP) (Fig. 1C; Passage 0). Nevertheless, these OSKM+LT induced biPSC colonies could not be sustained. With picking and passaging colonies using stem cell (SC) medium on mouse embryonic fibroblast (MEF) feeders, their morphology rapidly changed by Passage 2, and appeared flattened with individual cells being discernible and a weaker ALP positive intensity by Passage 3 (Fig. 1C; Passage 3). These colonies could not be maintained beyond Passage 3 or 4, and were lost without any structural aggregation and any residual cells in the locale became ALP negative.

### TGFβ signaling is prominently disrupted in the bovine blastocyst ICM

**Fig. 2.**
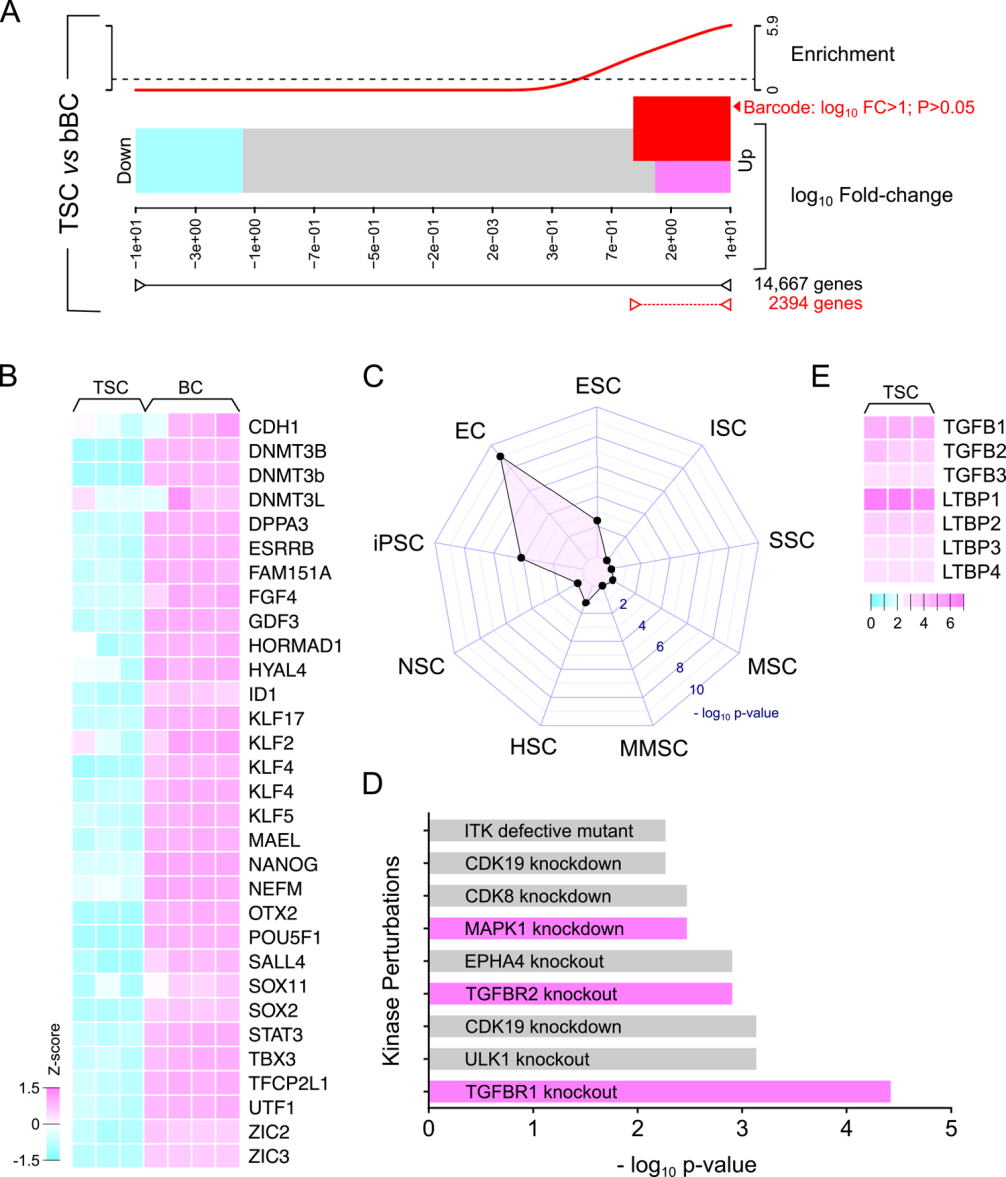
**Functional analysis of signal transduction in the bovine inner cell mass.** (A) Deducting transcriptome expression of bovine trophoblast stem cells (TSCs) from Day 8 blastocysts to enrich for genes expressed in the inner cell mass. Of the 14,667 genes in the transcriptome, 2394 genes were barcoded as upregulated in the bovine blastocyst (bBC) (log_10_ fold change >1.0 and *P*>0.05) and were separated for subsequent signal transduction analysis. (B) Heatmap showing that expression of conserved transcripts associated with pluripotency across different species were among these 2394 genes selected from the blastocyst transcriptome. (C) StemChecker identified transcriptional regulatory programs in the 2394 genes that were associated with and significant for embryonal carcinoma cells, iPSCs and ESCs. EC, embryonal carcinoma; ESC, embryonic stem cell; HSC, hematopoietic stem cell; iPSC, induced pluripotent stem cell; ISC, intestinal stem cell; MMSC, mammary stem cell; MSC, mesenchymal stem cell; NSC, neural stem cell; SSC, spermatogonial stem cell. (D) Analysis of kinase-based signaling in the 2394 upregulated genes indicated that TGFβ-mediated signaling was actively suppressed in the bovine inner cell mass. (D) Examination of transcripts in bovine TSCs showed that TGFβ1, TGFβ2 and TGFβ3 are expressed, and could be part of the blastocoel fluid. (E) Together with TGFβ, TSCs also expressed high levels of latent TGFβ-binding proteins (LTBPs), providing indirect evidence that TGFβ levels might be highly regulated around the developing inner cell mass.

In quantitative comparison of the transcriptome between early bovine blastocysts and undifferentiated TSCs, we were able to delineate gene expression specific to the ICM (Fig. 2A; Table S1). Within the cut-off value (log_10_ fold change >1 and *P*<0.05), we identified a list of 2394 genes possibly linked to the ICM. Inspecting known pluripotency-specific markers across different species revealed that this delineated gene list was highly enriched in expression of genes restricted to pluripotent cells (Fig. 2B). Analysis of this gene list for overlap with the gene signature of stem cells also showed significant enrichment of transcripts associated with ESCs, iPSCs and embryonal carcinoma cells (Fig. 2C). In kinase perturbation analysis of this gene list to discover signaling pathways suppressed in the blastocyst ICM, we uncovered that transforming growth factor β (TGFβ) signaling via TGFβ receptors (TGFBR1 and TGFBR2) were actively suppressed and highly significant (Fig. 2D). Trophectoderm/TSCs that contribute to the blastocoel microenvironment were found to express TGFβ1, TGFβ2 and TGFβ3, concurrently with high levels of latent TGFβ-binding proteins (LTBPs; Fig. 2E).

### Inhibition of TGFβ/activin/nodal pathway supports robust biPSC sustenance

**Fig. 3.**
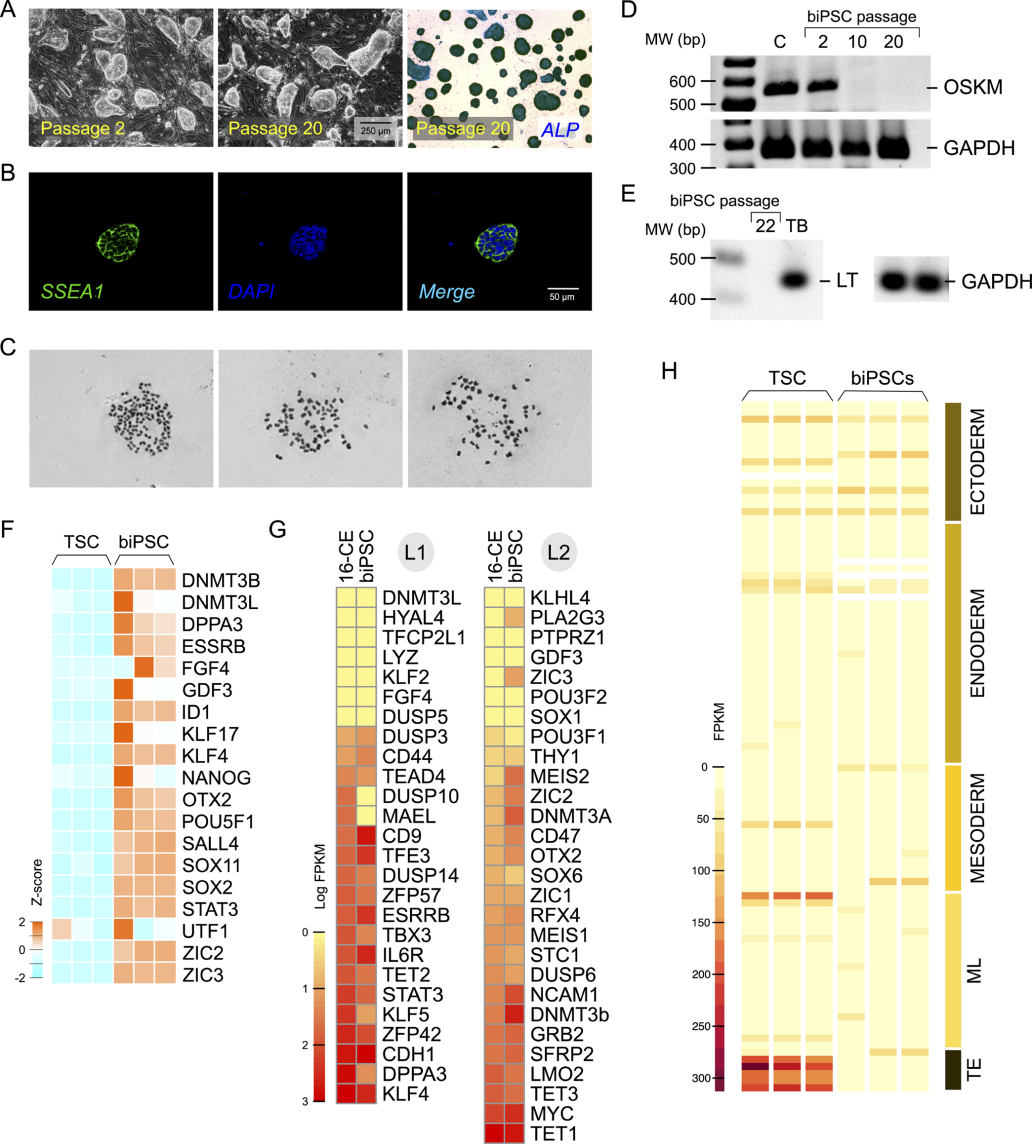
**Inhibition of TGFβ/activin/nodal in addition to GSK3β, MEK1/2 supports pluripotent self-renewal and long-term culture biPSCs.** (A) Representative images of biPSCs passaged in GMTi medium over an extended period of time. Colony morphology aligned with naïve PSCs as described for mice and humans and was strictly preserved in GMTi medium without any change indicative of differentiation. (B) Reprogrammed biPSCs were consistently SSEA1 positive across different passages (Passage 20 shown). (C) RT-PCR analysis of OSKM transgenes showed that expression was sustained in early passages (Passage 2) and silenced in later passages (Passage 10 and 20). (D) RT-PCR analysis of LT gene expression in biPSC (passage 20) also showed complete silencing. (E) Representative chromosomal spreads from individual biPSCs from passage 22 showing 60 chromosomes. (F) Gene expression profiling of biPSCs (performed at Passage 8) indicated endogenous activation of pluripotency-associated genes compared to expression seen in primary TSCs. (G) Expression levels of genes defined in studies on other species as associated with naïve (L1) or primed (L2) pluripotency, compared between 16-cell-stage bovine embryos (16-CEs) and biPSCs, indicated a mostly similar pattern of expression. (H) Genes associated with trophectoderm (TE), mixed lineages (ML), endoderm, mesoderm and ectoderm were low to no expression in biPSCs indicating an undifferentiated state. TE-restricted gene expression was observed only in TSCs. The full gene list used for this panel is included in Table S1.

Use of GMTi medium that contains inhibitor A83–01 (Tojo et al., 2005) to block downstream TGFβ signaling via SMADs, together with inhibitors for GSK3β and MEK1/2, supported robust self-renewal and repeated passages in sustaining cultures of biPSCs (Fig. 3A). Colonies showed a sustained morphology indicative of naïve pluripotency with little change from the time of dedifferentiation, with rounded edges and individual cells not being discernible (Fig. 3A). Colonies consistently stained positive for SSEA1 (Fig. 3B). After 22 repeated passages, clonal populations of biPSCs were found to have a normal karyotype (chromosomes 2n=60; Fig. 3C). Exogenous OSKM delivered by the STEMCCA vector was silenced in biPSCs in the later passages (Fig. 3D). Expression of LT was also silenced in biPSCs examined at later passages (Fig. 3E). Gene expression profiling showed upregulation of endogenous pluripotency-associated genes in biPSCs (Fig. 3F). Comparing expression of genes previously associated with naïve or primed pluripotency between 16-CEs and biPSCs indicated a generally similar pattern of expression (Fig. 3G). Gene expression specific to the trophectoderm (TE), and differentiation of the endoderm, mesoderm and ectoderm, were not observed in biPSCs (Fig. 3H; Table S1).

**Fig. 4.**
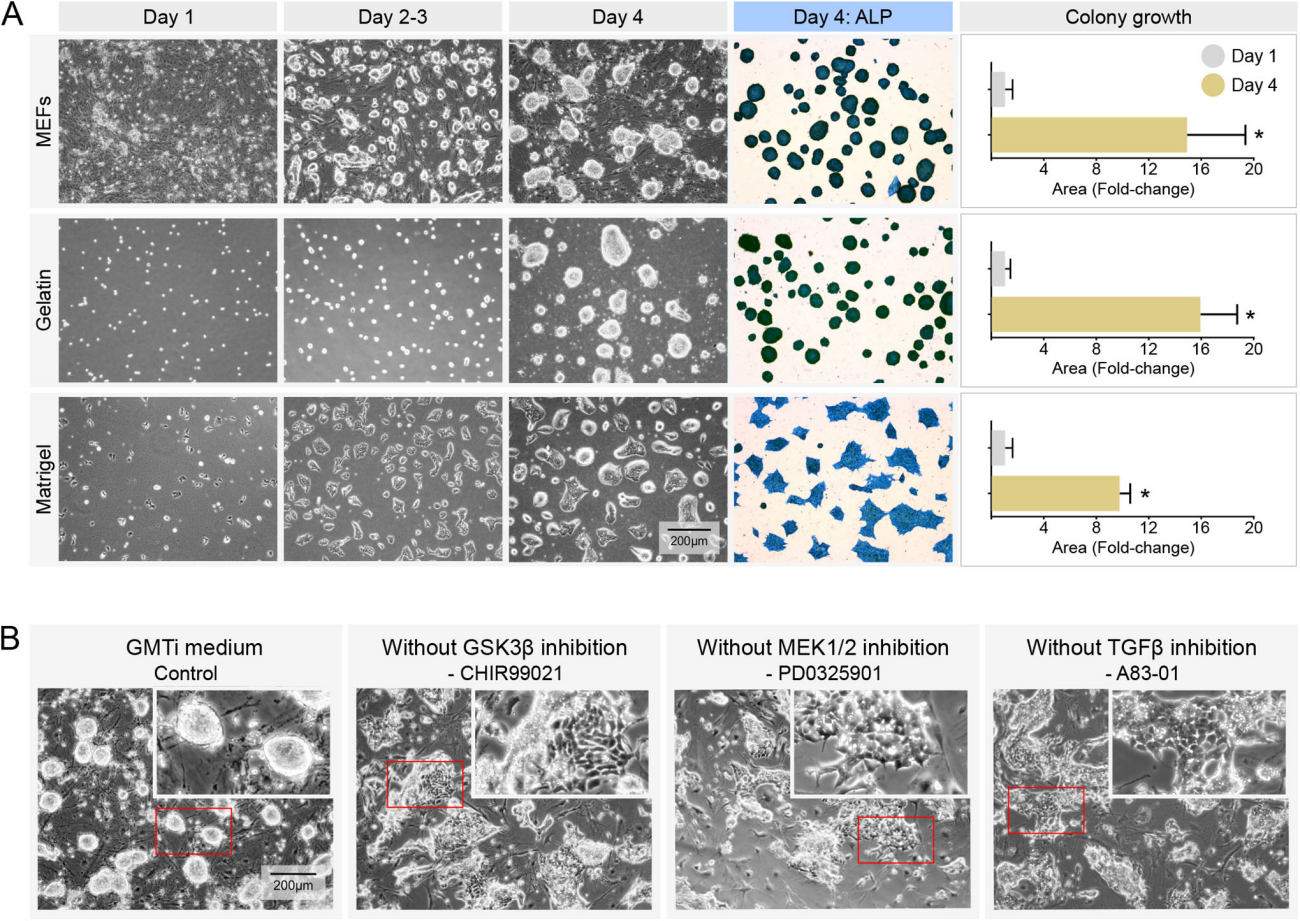
**Indispensable roles for GSK3β, MEK1/2 and TGFβ/activin/nodal inhibitors in biPSC pluripotency sustenance.** (A) Use of GMTi medium allowed culture of biPSCs on different cell-free surfaces such as gelatin and Matrigel^®^, without any effect on pluripotency sustenance. Representative images and quantification of size indicative of self-renewal and expansion of biPSCs over a period of 4 days. Rapid proliferation could be documented by average colony size increase of 10- to 16-fold over a period of 4 days (*n*=500-700 colonies/group; *P<0.001). (B) Including all three – GSK3β inhibitor (CHIR99021), MEK1/2 inhibitor (PD0325901) and TGFβ/activin/nodal inhibitor (A83–01) – is essential for sustaining the biPSC morphology and pluripotency. Eliminating one of the above three inhibitors in biPSC cultures resulted in differentiation. Representative images showing the effect of removing CHIR99021, PD0325901 or A83–01 in biPSC cultures; detrimental effects to colony morphology consistent with differentiation can be noted within 48 h of removal.

Culturing biPSC cells with GMTi medium allowed cells to grow in different cell-free substrates (Fig. 4A). They maintained a strong ALP-positive characteristic with no tendency toward differentiation. Rapid proliferation could be detected by average colony size increases by ∼15-fold, 16-fold and 10-fold in a period of 4 days when grown on iMEFs, gelatin and Matrigel^®^, respectively. Experiments excluding single inhibitors to GSK3β (CHIR99021), MEK1/2 (PD0325901) or TGFβ/activin/nodal (A83–01) indicated that all three are necessary to sustain pluripotency with colony morphology indicative of naïve cells. Removal of even one of these three inhibitors in biPSC cultures resulted in differentiation based on shifts to cell morphology that could be noted within 48 h (Fig. 4B). Beyond 48 h, cells showed complete spontaneous differentiation (data not shown).

### biPSCs differentiate into tissues of the three germ layers

**Fig. 5.**
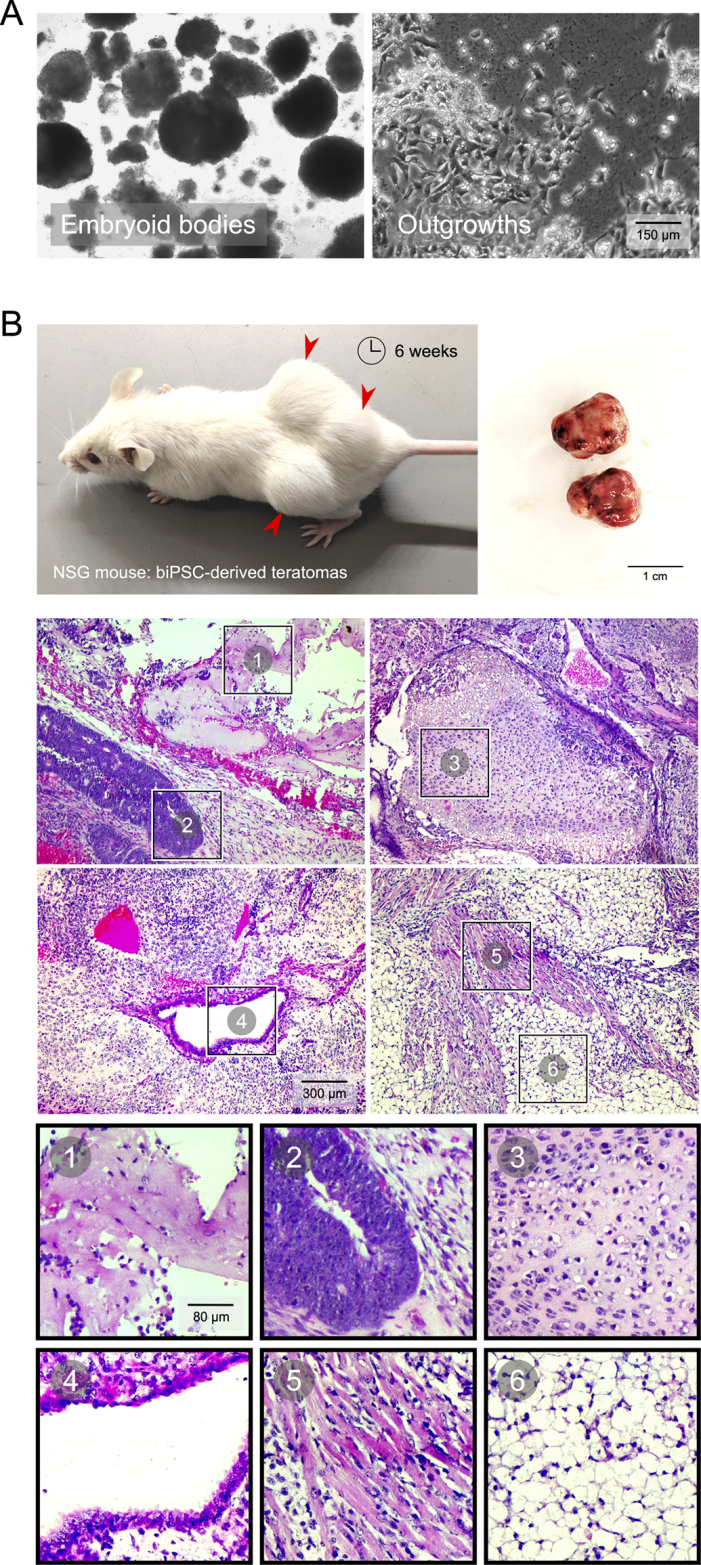
**Pluripotent biPSCs readily differentiate to cells of the three germ layers.** (A) Embryoid bodies could be generated from biPSC cultures with removal of inhibitors and supplementing serum. Representative images show embryoid bodies from 5 days of culture; outgrowths from these embryoid bodies when plated in gelatin-coated dishes presented a diversity of differentiated cell with distinctive morphologies. (B) Top left: subcutaneous introduction of biPSCs in immunodeficient NSG mice resulted in teratoma formation, with significant growth observed by 6 weeks. Top right: teratomas collected measured more than ∼1 cm in diameter. Bottom: representative images of Hematoxylin and Eosin-stained histological sections of teratomas showing differentiation of biPSCs into the three different germ layers. Four panels show different regions within teratoma sections with insets pointing to: (1) bone tissue (mesoderm), (2) neural tube/crest (ectoderm), (3) hyaline cartilage (mesoderm), (4) ciliated respiratory epithelium (endoderm), (5) cardiomyocytes (mesoderm) and (6) adipocytes (mesoderm).

Differentiation of biPSCs to embryoid bodies could be attained *in vitro* with suspension cultures without providing the inhibitors for self-renewal (Fig. 5A). These embryoid bodies were viable and generated outgrowths of differentiated cells when plated in cell culture-treated dishes. Subcutaneous grafting of biPSCs suspended in Matrigel^®^ into immunodeficient NSG mice resulted in prominent teratoma growth by 6 weeks (Fig. 5B). Teratoma sizes per injection site were ∼1 cm in diameter. Histological analysis of teratomas indicated the presence of different tissue types representing ectoderm, mesoderm and endoderm differentiation of biPSCs.

### Transcriptome uncovers regulatory machinery associated with bovine pluripotency

**Fig. 6.**
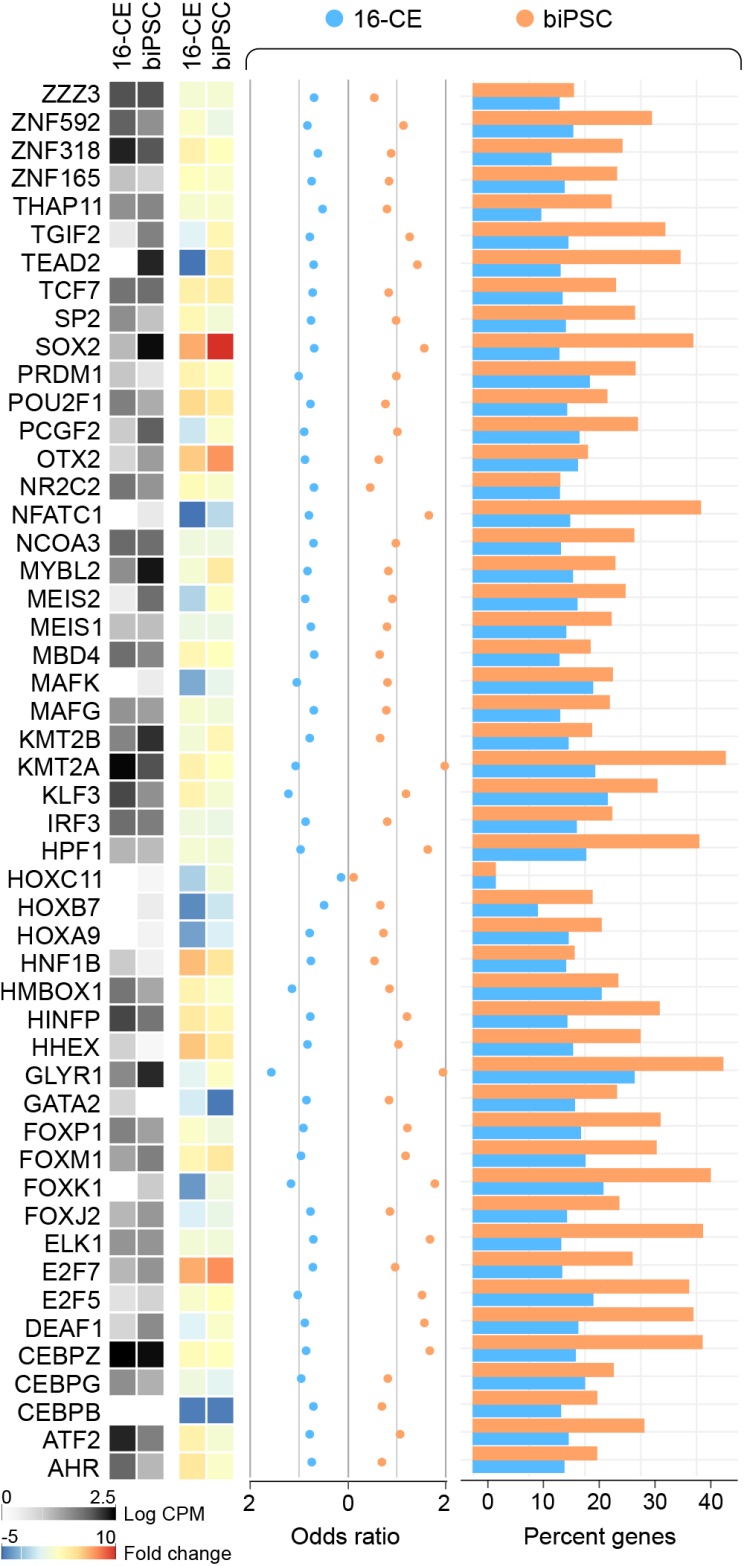
**Core transcription factors contributing to the phenotypic homeostasis of the bovid pluripotent state.** Systems biology investigation into active transcription enrichment that accounts for the majority of gene expression in 16-CEs and biPSCs indicated a consistent repertoire of significant factors between these two groups representing the bovid pluripotent state. Data represent expression levels for the identified transcription factors in 16-CEs and biPSCs [as counts per million (CPM) and fold change compared to fibroblasts], the strength of association (odds ratio) and the percentage targets detected in the analysis.

Analysis of active transcription that captures the majority of gene expression seen in biPSCs indicated a repertoire of 50 prominent factors between 16-CEs and biPSCs that dictated the phenotypic state (Fig. 6). This chromatin immunoprecipitation enrichment analysis (ChEA) indicated a core level of commonality in gene regulation indirectly informing of the chromatin state (not strictly linked to pluripotency). Although all the predicted transcriptional regulators were expressed in both biPSCs and 16-CEs, in quantitative comparisons with fibroblasts, only ten transcription factors (SOX2, E2F7, HNF1B, OTX2, POU2F1, FOXM1, HHEX, TCF7, HINFP and ZNF318) were prominently upregulated >2-fold in both, indicating an exclusive phenotypic contribution of these factors to bovine pluripotent cells (available gene descriptions provided in Table S1).

**Fig. 7.**
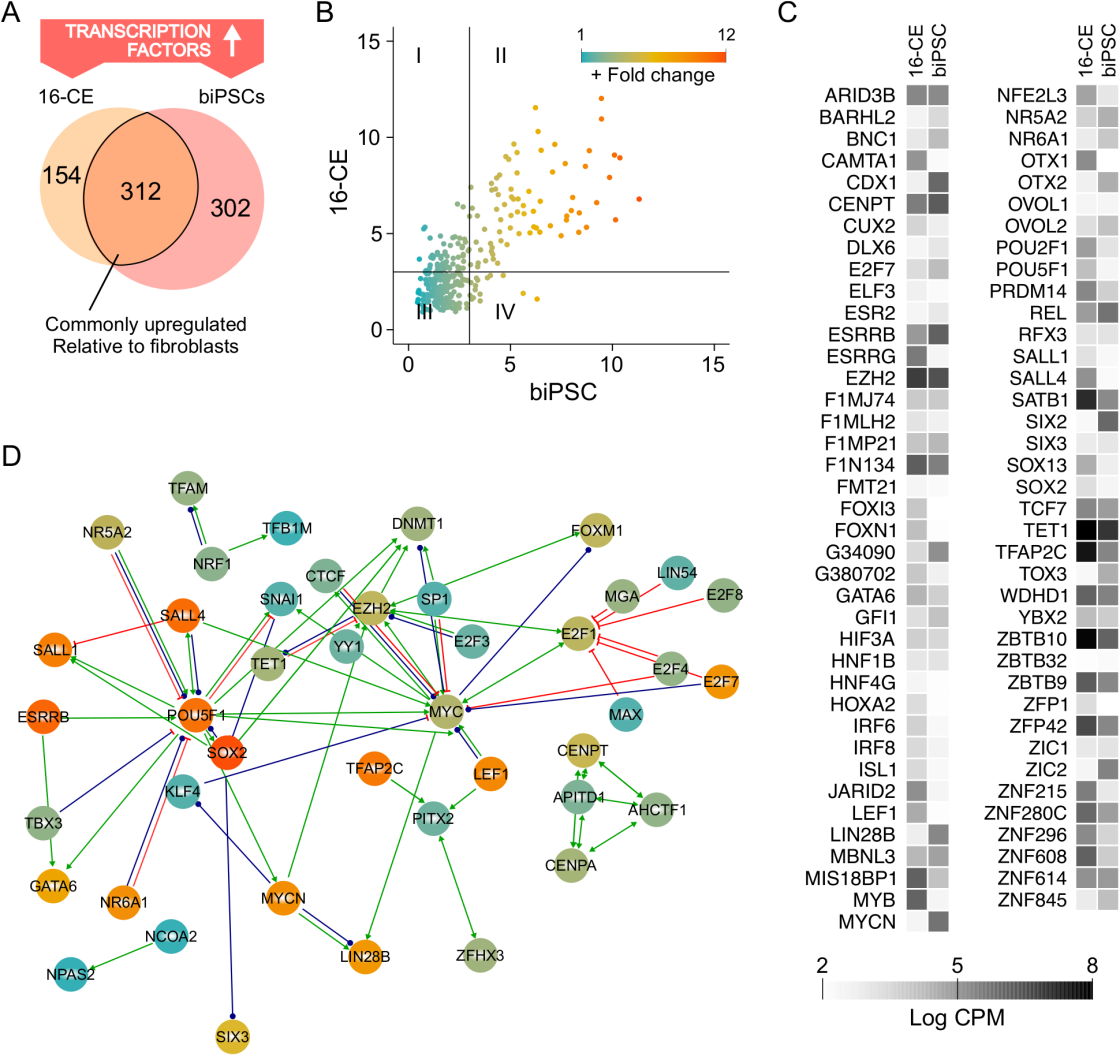
**Transcription regulator network associated with sustenance of bovid pluripotency.** (A) Comparison of transcription factors upregulated in the 16-CEs and biPSCs (fold change compared to fibroblasts) identified 312 common regulators. (B) Plotting the correlation of fold change across these common regulators between biPSCs and 16-CEs revealed 77 factors as highly expressed in both groups (quadrant II, with a >3-fold-change upregulation cutoff). (C) Expression levels (as CPM) for the 77 factors identified in quadrant II. (D) Constructing an interaction network map of transcriptional regulators common for 16-CEs and biPSCs disclosed the known regulatory mechanisms associated with the identified genes.

In separate analysis, comparing the full list of transcription factors that were upregulated relative to fibroblasts [false discovery rate (FDR) <0.05] between biPSCs and 16-CEs indicated 312 common factors (Fig. 7A). This represented an exhaustive list of all the transcriptional regulators likely involved in pluripotency (Table S1). Plotting relative abundance (fold change) between biPSCs and 16-CEs to visualize the most prominent regulators revealed 77 common factors that were >3-fold higher in both biPSCs and 16-CEs (quadrant II, Fig. 7B). Examining expression levels (CPM) of these 77 common regulators indicated a consistent range in the levels of baseline expression in both 16-CEs and biPSCs (Fig. 7C). This list contained numerous factors that are not previously associated with pluripotency, and factors that remain uncharacterized genes. Mapping for functional association in the common regulators uncovered what is known of the transcriptional framework associated with bovine pluripotency, which also indicated that the biPSCs and 16-CEs had fundamental similarities (Fig. 7D). In addition to containing all four reprogramming-capable genes (OSKM), the map identified known interactions with other factors associated with specific functions such as epigenetic modification/chromatin remodeling, transcriptional repression/activation, cell cycle regulation, X chromosome inactivation and estrogen signaling (Table S1). This overlap between biPSCs and 16-CEs indicates similarities in transcriptional regulation and/or epigenetic state for existing or in preparation for future functional states.

### Integrated algorithms reveal scope of extrinsic signal perception in bovine pluripotent stem cells (PSCs)

**Fig. 8.**
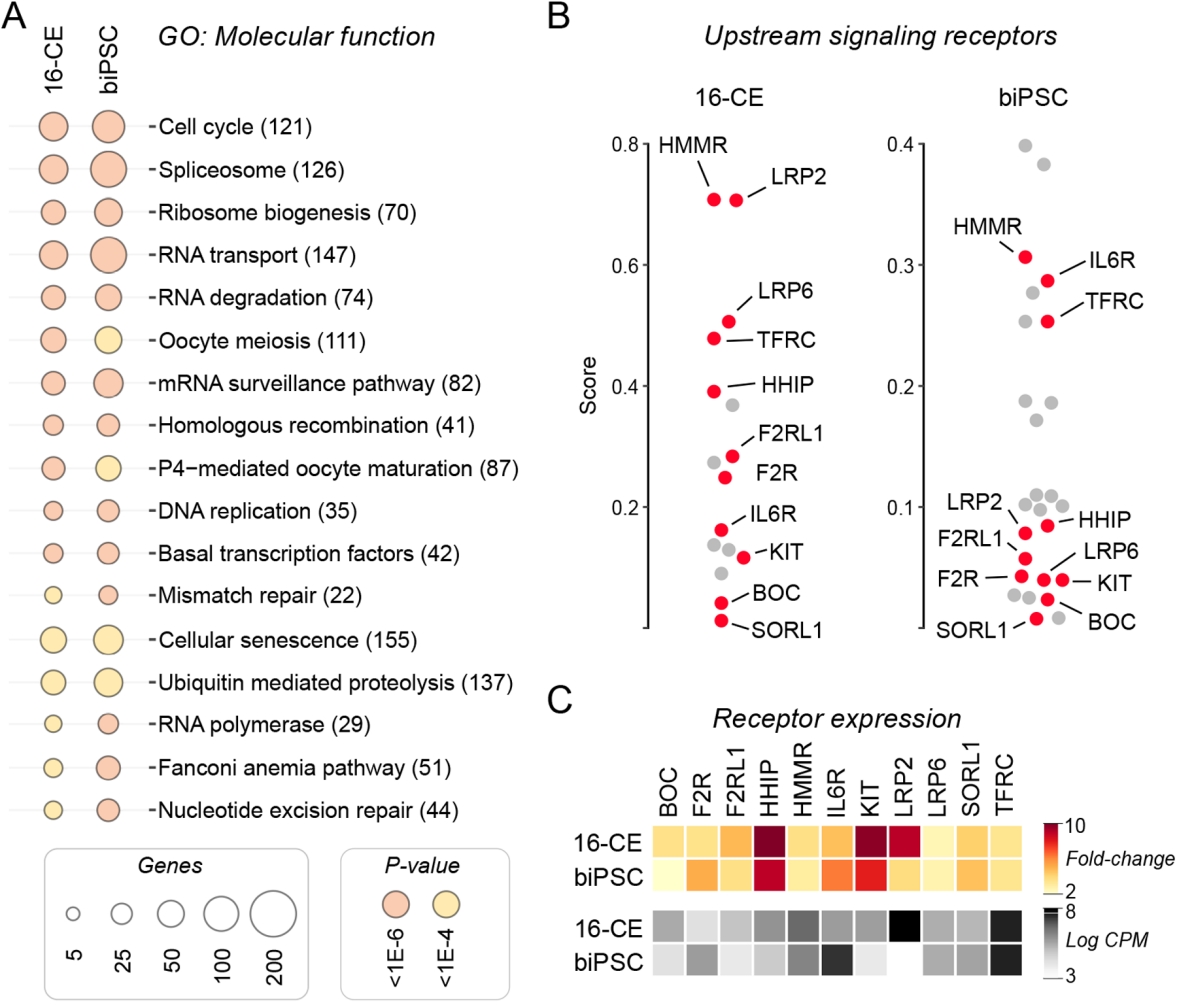
**Integrative expression-interaction analysis predicts cell surface receptors that regulate pluripotency signaling in bovids.** (A) Analysis of gene ontology (GO) under molecular function indicated common systems between 16-CEs and biPSCs in upregulated genes; comparisons to fibroblasts were evaluated independently and significant elements merged for 16-CEs and biPSCs. (B) Integrative analysis combining gene expression and protein–protein interactions predicted 11 common upstream membrane receptors that represent signaling in 16-CEs and biPSCs. (C) Expression levels for these 11 identified membrane receptors were highly expressed in both 16-CEs and biPSCs. Heatmaps show expression as CPM and fold change compared to fibroblasts.

Pathway analysis performed using genes upregulated in biPSCs and 16-CEs (in comparison to fibroblasts) indicated common enrichment to functional features (Fig. 8A). Beyond the basic cell maintenance and regulatory mechanisms, specific functional enrichment indicating rapid cell turnover and genome maintenance were uncovered: ribosome biogenesis, DNA replication, cellular senescence, oocyte meiosis, homologous recombination, mismatch repair and ribosome biogenesis. Examination for upstream receptor-mediated signaling revealed 11 surface membrane receptors common to both biPSCs and 16-CEs that capture signaling, and active transcriptional mechanisms that represent the bovine pluripotent state (Fig. 8B). Receptor for the leukemia inhibitory factor family member IL6 was part of this list together with several other receptors previously unassociated with pluripotency. Expression levels examined for these receptors indicate that they were all significantly upregulated in biPSCs and 16-CEs compared to fibroblasts (FDR >0.001; Fig. 8C). This corroborated that the identified receptors are specific and relevant to bovine pluripotent cells.

## DISCUSSION

In this report, we present the formula for generating naïve biPSCs with complete reprogramming to pluripotency, prolonged self-renewal capacity and silenced transgenes, a task that has remained a challenge despite numerous studies on this topic (Su et al., 2020). Using these cells, we have uncovered core characteristics of transcriptional regulation and signaling that defines the bovine pluripotent state, allowing comparative evaluation based on what is known in other species.

From previous work, we concluded that bovine fibroblasts might have a stable epigenome that makes them refractory to complete reprogramming; OSKM did not induce colony formation in bovine fibroblasts (Pillai et al., 2019b). This meant that even if pluripotency genes are active/induced, cells are fixed in a differentiated phenotype or readily revert to a differentiated phenotype, as large sections of the epigenome do not support the pluripotent self-renewal program. As reprogramming efficiency is positively correlated to the rate of complete reprogramming (Mikkelsen et al., 2008), we investigated an extra factor LT together with OSKM. It has been shown that addition of LT with OSKM shows dramatic improvement to the pace and reprogramming efficiency in human cells (Mali et al., 2008; Park et al., 2008a,b). Recently, it was shown that LT could help reprogram naked mole rat fibroblasts that were documented to be resistant to reprogramming by OSKM alone (Tan et al., 2017). Consistent with these reports, we found that LT significantly enhances reprogramming efficiency in cattle.

Albeit not explained on the basis of complete reprogramming, such effect has also been indicated in certain ruminants such as sheep (Bao et al., 2011) and goats (Ren et al., 2011). Linked to a variety of influences encompassing transcription and epigenetics, LT affects a gamut of cellular targets/processes (Ahuja et al., 2005). One of the prominent effects of LT reported in the literature is its interaction and inactivation of TP53 (p53) functions, and the retinoblastoma family of proteins (particularly RB), dysregulating pivotal checkpoints in cell cycle control (Ali and DeCaprio, 2001). Although knockdown of TP53 has been shown to increase reprogramming efficiency in murine and human cells (Kawamura et al., 2009; Zhao et al., 2008), we do not believe LT action in inducing biPSCs is via an effect on TP53, as similar knockdown of TP53 in bovine embryonic fibroblasts (BEFs) did not enhance reprogramming efficiency (Pillai et al., 2019b). The equilibrium guiding TP53 and RB activities are known to vary between species; in the naked mole rat, reprogramming block released by LT was identified as being due to RB rather than TP53 (Tan et al., 2017). It has been shown that RB stabilizes heterochromatin via interactions with H3K9 methylases (Ait-Si-Ali et al., 2004; Nielsen et al., 2001). In association, RB was demonstrated to restrict reprogramming in murine fibroblasts by maintaining a more repressive chromatin state (Kareta et al., 2015). In somatic cell nuclear transfer experiments using BEFs, it was identified that failure of H3K9 demethylation presented a block to nuclear reprogramming (Liu et al., 2018). In this context, it can be interpreted that the LT induced increase in biPSC reprogramming could be at least in part due to an effect on RB. Nevertheless, cells derived using OSKM+LT could not be sustained or passaged without spontaneous differentiation in culture using SC medium. At this point, it was unclear whether this was due to incomplete reprogramming or solely the lack of appropriate medium conditions for sustenance.

Studies using small-molecule inhibitors for MEK1/2 (PD0325901) and GSK3β (CHIR99021) have previously shown that they suspend biPSCs in a pluripotent state, but without the ability to proliferate (Huang et al., 2011). It has also been suggested that use of these same two inhibitors during the reprogramming process could yield biPSCs capable of self-renewal in culture, with added valproic acid (to inhibit histone deacetylation) and ascorbic acid (to promote histone and DNA demethylation) (Heo et al., 2015). Use of these inhibitors originated from studies that defined the naïve ‘ground state’ of mESCs, which demonstrated that inhibitors for MEK1/2, GSK3β and FGFR released pluripotency from the dependence of exogenous growth conditions (Ying et al., 2008). Concurrent use of N2 and B27 supplements could support serum-free bulk cultures of naïve mESCs (Ying et al., 2008). In contrast, FGFR inhibition has been shown not to be critical for sustaining naïve hESCs that could be maintained with inhibition of MEK1/2, GSK3β and PKC in the presence of hLIF (Guo et al., 2016). Notwithstanding, our attempts to use the above inhibitors with and without growth factors on OSKM+LT-reprogrammed cells did not support self-renewal and/or ability to passage colonies (not shown). Therefore, we turned to analysis of gene expression relevant to the bovine blastocyst ICM to learn more about pathway targets that may be exclusive for biPSCs.

Specific enrichment for signaling perturbation indicated TGFBR1 signaling as a top presentation in the bovine ICM gene expression. In murine iPSCs (miPSCs), use of TGFβ inhibitors has resulted in faster and more efficient induction of iPSCs; conversely addition of TGFβ has been shown to block reprogramming (Maherali and Hochedlinger, 2009). Subsequently, it was demonstrated that TGFβ inhibition supports pluripotency by reducing ERK phosphorylation in miPSCs (Tan et al., 2015). In contrast, it was demonstrated that TGFβ signaling is necessary for the maintenance of pluripotency in hESCs (James et al., 2005). Although these studies made it clear that TGFβ effects are not conserved in pluripotent cells across different species, following our analysis, we discovered that TGFβ inhibition coupled with MEK1/2 and GSK3β inhibition could support robust cultures of naïve biPSCs. As pluripotency in *in vivo* blastocysts is critically shaped by the trophectodermal contributions to the blastocoel fluid (Pillai et al., 2019a), we speculate that the LTBPs present minimize the levels of TGFβ available and restrict morphogenesis during pluripotent expansion. It is well known that TGFβ family of proteins (that include activin and nodal) are critical for specifying the body plan during metazoan development (Wu and Hill, 2009). During the period of our work, it was also revealed that a six-small-molecule cocktail that included a TGFβ inhibitor in combination with MAPK14, MAPK8, MAP2K1, GSK3α and BMP supports naïve porcine iPSC lines in the presence of both FGF2 and LIF (Yuan et al., 2019). In comparison, biPSC cultures were liberated from dependence on growth factors in GMTi medium, a significant step forward towards uncovering pluripotency regulation in bovids and ruminants.

Complete reprogramming and long-term sustenance have not been reproducibly achieved in previous attempts to generate biPSCs using OSKM factors alone. Colony morphology coupled to gene expression analysis indicated naïve-type biPSCs. We base this definition on the fact that pluripotency gene expression in these biPSCs closely reflects that observed in 16-CEs. In specifically examining genes defined in studies on other species as associated with naïve or primed pluripotency, we find that expression in the 16-CEs representative of bovine naïve cells is novel and not confined to this bifurcated pattern. These results present the molecular signature of naïve cells in ruminant pluripotency that is also observed in the generated biPSCs in GMTi medium. We did not encounter any aberrant reprogramming into the trophoblast lineage, although this has been reported to occur in bovine cells transduced with OSKM factors (Kawaguchi et al., 2016; Talbot et al., 2017). This perhaps suggests that OSKM+LT resulted in complete reprogramming, followed by sustenance that ultimately silenced the exogenous OSKM and LT expression. The biPSCs cultured across multiple passages, and expanded under feeder-free conditions, were robust in generating embryoid bodies and readily differentiated into teratomas that were composed of ectodermal, mesodermal and endodermal lineages. The ability to sustain the biPSCs provided, for the first time, an opportunity to rigorously examine the bona fide transcriptional regulation and pathways associated with pluripotency in cattle.

In evaluating the transcriptional contribution to the en masse phenotype of bovine pluripotency, we identified ten enriched factors that were consistently upregulated in biPSCs and 16-CEs. Of these, SOX2 has been well known for its role in pluripotency sustenance across different species (Avilion et al., 2003; Rodda et al., 2005) and is a component of the reprogramming factors used for iPSC generation (Takahashi and Yamanaka, 2006). Similarly, POU2F1/OCT1, a paralog of POU5F1/OCT4 that shares binding specificity by heterodimerization (Fletcher et al., 1987; Tomilin et al., 2000), was found to be a substantial contributor. POU2F1 is also known to interact with other cofactors, suggesting a larger repertoire of targets and distinct specificities to this paralog (Shakya et al., 2011; Tomilin et al., 2000). Both POU2F1 and POU5F1 are known to interact with SOX2, albeit with differential activation properties (Ambrosetti et al., 1997). Involvement of OTX2 has been linked to maintenance of the metastable ESC state by opposing self-renewal and predisposing cells to differentiation (Acampora et al., 2013). Function of TCF7 as a binding partner of beta-catenin, the core factor involved in the transcriptional output of WNT signaling is well known (Cadigan and Waterman, 2012; Mao and Byers, 2011). The mechanism of GSK3β inhibition used to maintain pluripotency (Sato et al., 2004), as used in this study to maintain biPSCs, is via promoting beta-catenin targets. The atypical E2F7 responsible for transcriptional repression at E2F sites (Carvajal et al., 2012; Di Stefano et al., 2003; Liu et al., 2013) also regulated the expression landscape. The contribution of HNF1B and ZNF318 remains unknown, with no prior studies to provide an interpretation of possible function.

In parallel, expression-based cut-off identifying 77 transcription regulators highly consistent between biPSCs and 16-CEs showed the specific transcriptional regulation underlying bovine pluripotency. This list included transcription regulators already known to be associated with pluripotency, together with several factors previously not associated with pluripotency, and few uncharacterized genes in cattle. This list forms a core resource for future investigations into divergent aspects of bovine pluripotency regulation. Mapping known elements from the commonly upregulated list showed specific nucleation in the transcriptional network indicating core influences of OCT4/POU5F1, SOX2, MYC, E2F1and EZH2.

The algorithm identifying the 11 common membrane receptors from corresponding integration of gene expression and signaling could be considered as rigorous, as all these receptors were highly expressed in both biPSCs and 16-CEs, indicating high relevance in pluripotency. This list included known elements such as IL6R, from which, downstream signaling via STAT3 is known to sustain pluripotency in mESCs (Nichols et al., 1994). LIF, an IL6 family member, is long known to support pluripotency signaling in mESCs (Smith et al., 1988; Williams et al., 1988). Recently, IL6 treatment has been shown to increase cell numbers of the ICM in bovine blastocysts (Wooldridge and Ealy, 2019), via a direct or indirect mitogenic effect on bovine pluripotent cells. However, excluding LIF or IL6 from the GMTi medium did not negatively affect biPSCs at least over a few passages, suggesting that with GSK3β, MEK1/2 and TGFβ/activin/nodal inhibition, IL6R-based signaling does not add to pluripotency sustenance as indicated by cell morphology. Not to be discounted yet is that biomimicry by providing IL6 in long-term cultures could buttress endogenous mechanisms in long-term sustenance, as IL6R is indeed expressed in both biPSCs and 16-CEs. Another element, KIT, a receptor tyrosine kinase, is known to be expressed in ESCs (Palmqvist et al., 2005). It has been demonstrated that KIT inhibition can affect both self-renewal and survival of differentiating cells (Bashamboo et al., 2006; Fraser et al., 2013). Receptor HMMR, and potential for signaling mediated by hyaluronan, has not been previously dissected in pluripotency. However, it was reported that hyaluronan-gelatin hydrogels could maintain miPSCs and human iPSCs (Liu et al., 2012). Recently, it has been observed that highly sulfated hyaluronic acid could maintain primed human iPSCs by promoting FGF2-ERK signaling, even in the absence of recombinant FGF2 (Miura et al., 2019). Although expressed, Sonic Hedgehog (SHH) signaling in human and mouse PSCs has not been linked to self-renewal but only in differentiation toward the neuroectodermal lineage by some studies (Lau et al., 2019; Wu et al., 2010). In contrast, one study in mESCs suggests that SHH-mediated GLI1 activation and phosphorylation of EGFR supports self-renewal (Heo et al., 2007). In subsequent studies, a more intricate relationship has been established balancing pluripotency and differentiation in that NANOG interacts with GLI1, providing negative feedback, permissive only to PSC-specific regulation of SHH signaling (Li et al., 2016). Our identification of HHIP (a member of the hedgehog interacting protein family), and BOC (a member of the immunoglobulin/fibronectin type III repeat family), both components of the cell surface SHH receptor complex (Izzi et al., 2011), supports a possible role for SHH signaling in bovine pluripotency. Members of the low-density lipoprotein receptor family widely known to be involved in receptor-mediated endocytosis and associated endosomal sorting of lipoprotein and other protein ligands (LRP2, LRP6 and SORL1) were also identified. Of these, LRP6 has been shown to be a component of the WNT complex that triggers beta-catenin signaling (Cselenyi et al., 2008); LRP2 has been shown to act as an auxiliary SHH receptor by increasing signaling capacity (Christ et al., 2012); and SORL1 has been shown to be integrally involved in IL6 signaling, specifically promoting capacity for soluble IL6R or *trans* signaling as opposed to the classic *cis* signaling (Larsen and Petersen, 2017). Transferrin receptor (TFRC), widely known for iron acquisition by all mammalian cells (Dautry Varsat et al., 1983; Jandl et al., 1959), was also identified; iron uptake has been recently shown to promote WNT/β-catenin signaling (Mandala et al., 2020; Song et al., 2011). Protease-activated G-protein coupled receptors (F2R and F2RL1) have not previously been studied in PSCs. There are a variety of known signaling mechanisms supported by these receptors (Heuberger and Schuepbach, 2019), the relevance of which requires additional investigation. Collectively, similarities presented in these signaling receptors between the 16-CEs and biPSCs not only indicate its authenticity, but also presents novel information regarding extracellular signaling mechanisms/mediators that might find critical roles in sustaining the bovine pluripotent state.

In developing these methods and mechanisms, we find it difficult to reconcile with recent studies which suggest that inhibition of WNT signaling (using IWR1) is crucial for the derivation and propagation of a primed form of bESCs (Bogliotti et al., 2018) and bovine expanded potential stem cells (EPSCs) (Zhao et al., 2021) [the latter was during the time this manuscript was under review]. However, the distinctions we find do support the notion that maturation through a continuum of pluripotent states *in vivo* can be captured in the form of different stable transitional states *in vitro* (Morgani et al., 2017). WNT/β-catenin signaling has been reported to be critically calibrated in early development and pluripotency (Zhang et al., 2013). In naïve murine PSCs, repressing WNT signaling induced differentiation towards a primed epiblast stem cell (EpiSC) state (ten Berge et al., 2011), and in the primed state, WNT activation (using CHIR99021) can result in intermediate pluripotent stem cells (intPSCs) that exhibit characteristics of both ESCs and EpiSCs (Tsukiyama and Ohinata, 2014). Although it is difficult to extrapolate these mechanisms to bovine PSCs, it is plausible that the primed form of bESCs captured using IWR1 (Bogliotti et al., 2018), and bovine EPSCs captured using both IWR1 and CHIR99021 (Zhao et al., 2021), both derived from blastocysts, represent stable transitional states.

In conclusion, we have successfully established completely reprogrammed naïve biPSC lines that show core parallels to 16-CEs. In addition to opening up possibilities for comparative studies on the basis of pluripotency regulation in a species that has baffled scientists for decades, we present a complete tool for advancing reproduction and biotechnology applications in an agriculturally important species.

## MATERIALS AND METHODS

### Primary cells and culture

Bovine embryos (at 35-45 days in development) were collected from an abattoir (Cargill, Wyalusing, PA, USA) for culturing BEFs as previously described (Pillai et al., 2019b). Embryos were first decapitated and eviscerated before mincing into small pieces less than 1 mm^3^, then plated for culture in fibroblast medium [Dulbecco's minimal essential medium (DMEM) with high glucose containing 10% fetal bovine serum (FBS), 1% non-essential amino acids supplement and penicillin-streptomycin]. Cells were allowed to grow in a 37°C humidified incubator under an atmosphere of 5% CO_2_. Once cells were confluent, they were passaged twice for expansion and frozen aliquots prepared for experiments. Irradiated mouse embryonic fibroblast (iMEF) feeders were prepared from cells cultured from embryonic day 13.5 mouse embryos as previously described (Pillai et al., 2019b).

### Viral vectors and induction of reprogramming

The human STEMCCA polycistronic lentiviral reprograming vector (Sommer et al., 2009), a lentiviral simian vacuolating virus 40 large T antigen vector (Mali et al., 2008), and bovine Nanog, previously generated by gene synthesis (Pillai et al., 2019b) and inserted into a lentiviral backbone (pLenti-EF1α), were used. In brief, 293T cells were co-transfected with gene inserts and helper plasmids that encode for lentiviral Gag, Pol, and Env proteins as previously described (Pillai et al., 2019b). Virus-containing supernatants were collected at 48 and 72 h, and pooled and passed through a 0.45 µm syringe filter before use in infecting BEFs. A green fluorescent protein (GFP) expressing pLenti-EF1α-GFP vector was used to package control lentiviruses to monitor packaging and infection efficiency. The method timeline is as indicated in Fig. 1A: BEFs were infected using STEMCCA in fibroblast medium supplemented with 6 µg/ml Polybrene (Sigma-Aldrich) for 24 h. Seven days after infection, BEFs were collected by trypsinization (0.5% Trypsin EDTA, Millipore) and plated on iMEFs at a density of 2.5×10^4^ cells/cm^2^. After cell attachment the next day, the medium was changed to SC medium [fibroblast medium containing 0.1 mM β-mercaptoethanol, 10 ng/ml human LIF (Millipore) and 20 ng/ml human FGF2 (Peprotech)]. To assess reprogramming efficiency, plates were collected at day 25 for staining and quantification of colonies. For propagation, individual colonies were manually picked, dissociated into single cells using TrypLE^TM^ (Thermo Fisher Scientific) and plated on iMEFs in SC medium. Subsequent passages were performed by identical picking, dissociation and plating of individual colonies on iMEFs. Passaging was continued as long as cells/colonies could be identified and propagated. All cultures were maintained in a humidified incubator at 37°C under an atmosphere of 5% CO_2_. Images were acquired using an inverted microscope (DMIL-LED, Leica) and a high-definition camera (MC190HD, Leica).

### ALP staining

Labeling for ALP activity was performed using a kit (Vector Blue AP Substrate Kit) to visualize and quantify biPSC reprogramming progress. Reagents were added according to manufacturer instructions to 10 ml Tris-HCl, pH 8.5 buffer, and sufficient solution was added to wells containing cells and incubated for 30 min at 37°C under an atmosphere of 5% CO_2_. Entire plates were imaged for quantifying biPSC colonies for estimating reprogramming efficiency.

### Analysis of signaling in the bovine ICM

RNA sequencing (RNA-seq) was performed using bovine blastocysts (day 7 after *in vitro* fertilization). *In vitro* embryo production was as previously described (Pillai et al., 2019a). Three independent batches of blastocysts produced were used for sequencing (∼120 blastocysts/group). In brief, total RNA was extracted from three independent groups of blastocysts using an RNAqueous Micro Total RNA Isolation Kit (Thermo Fisher Scientific). Integrity of total RNA was checked using a Bioanalyzer 2100 (Agilent Technologies). Poly(A) capture was used to isolate mRNA. Fragmentation and cDNA library construction were performed using a TruSeq stranded total RNA sample preparation kit (Illumina). ﻿Three samples with unique bar code sequences were pooled for sequencing by synthesis to obtain short single reads on a HiSeq4000 (Illumina). Raw reads were subjected to quality control checks using FastQC tool (Babraham Bioinformatics). Reads were mapped to the bovine genome (ARS UCD 1.2) using bovine genome annotation file (Ensembl) using spliced transcripts alignment to a reference/STAR (Dobin and Gingeras, 2015). Comparisons were done to identify differentially expressed genes between blastocysts and undifferentiated TSCs [previously published by our group, Gene Expression Omnibus (GEO) GSE122418 (Pillai et al., 2019a)] using R package EdgeR (Robinson et al., 2010). Linear modeling, differential expression and a barcode plot visualization tool, Limma (Ritchie et al., 2015), were used for enrichment and to examine genes that were significantly upregulated in the blastocysts compared to TSCs, to delineate gene expression associated with the ICM. This gene list was then examined for known pluripotency-associated factors and subjected to analysis of overlap with gene signatures associated with stem cells using StemChecker (SysBiolab^©^) (Pinto et al., 2015). After the above validation, the gene list was subjected to enrichment analysis using the ESCAPE database with the Enrichr tool (Xu et al., 2013), to identify specific ‘Kinase Perturbations’ in the ICM signaling pathways. Complete embryo RNA-seq datasets are available through NCBI GEO (GSE169674).

### Sustenance of biPSCs using specific pathway inhibition

In iterative testing of methods compiled from previous published results (Pillai et al., 2019b) together with integrated data mining for signal transduction, we established the GMTi medium that contained inhibitors for GSK3β, MEK1/2 and TGFβ/activin/nodal (DMEM/F12 containing N2 supplement, B–27 supplement, 1% non-essential amino acids supplement, 1% penicillin-streptomycin, 0.1 mM β-mercaptoethanol, 1.5 μM CHIR99021, 1 μM PD0325901, 0.5 μM A83–01, and 20 ng/ml hLIF or 20 ng/ml hIL6). The biPSC colonies emerging from reprogramming trials were manually picked, dissociated into single cells using TrypLE™ (Thermo Fisher Scientific) and plated on iMEFs in GMTi medium. For passaging, confluent cultures of biPSCs were rinsed once with PBS and incubated with TrypLE for 5 min. Cells were then collected in fibroblast medium and centrifuged at 200×***g*** for 5 min. The pellet was then resuspended in GMTi medium for plating on iMEFs. Passaging of biPSCs was performed repeatedly (every 3-4 days), with concurrent examination and imaging of morphology, growth characteristics and expression of PSC markers. Growth/expansion was indirectly estimated by measuring the surface area of biPSC colonies using ImageJ (Schneider et al., 2012). Base medium conditions lacking individual inhibitors for GSK3β, MEK1/2 or TGFβ/activin/nodal were also prepared and tested for their ability to sustain pluripotent cultures. All cultures were maintained in a humidified incubator at 37°C under an atmosphere of 5% CO_2_.

### Feeder-free culture of biPSCs

Cell culture dishes coated with gelatin or Matrigel^®^ were used for feeder-free cultures. For gelatin coating, culture dishes were incubated with 0.2% gelatin from porcine skin (Type A, MilliporeSigma) for 24 h in a 37°C humidified incubator, solution aspirated and the gelatin film allowed to air dry before use. For Matrigel^®^ (Corning) coating, culture dishes were incubated with Matrigel^®^ (diluted in cold DMEM/F12, 9 µg/cm^2^) for 2 h in a 37°C humidified incubator, rinsed once with DMEM/F12 before immediate use. To avoid iMEFs when transitioning to feeder-free cultures, biPSC colonies were picked and dissociated into single cells using TrypLE and plated in GMTi medium. Subsequent passages were as described for propagation on iMEFs. Cells were examined and imaged for morphology, growth characteristics and expression of PSC markers. All cultures were maintained in a humidified incubator at 37°C under an atmosphere of 5% CO_2_.

### Immunocytochemistry

Bovine iPSCs were grown on coverslips seeded with irradiated MEF feeders and fixed with 4% formaldehyde. Cells were then permeabilized with 0.1% Triton X-100 in PBS for 1 min and blocked using 5% normal goat serum for 30 min. Coverslips were subsequently incubated with antibodies against SSEA1, 4 and 3 (1:200 dilution; Iowa Hybridoma Bank) for 1 h. Coverslips were then washed three times using PBS, incubated with Alexa Fluor-conjugated anti-mouse Fab’ fragments for 30 min, washed again with PBS, and counterstained/mounted with 4′,6-diamidino-2-phenylindole (DAPI) containing Prolong Gold reagent (Life Technologies, Carlsbad, CA, USA). Images were acquired using an inverted microscope (DMI 3000, Leica) using a cooled monochromatic camera (DFC365FX, Leica).

### Karyotyping

Bovine iPSCs cultured without feeders were treated with 0.1 µg/ml mitotic arrestant, colcemid (Life Technologies) for 16 h. The cells were then rinsed with PBS, and trypsinized to obtain a single cell suspension and pelleted by centrifugation at 100 ***g*** for 5 min. The cells were then resuspended in 5 ml hypotonic solution (0.56% KCl), incubated at 37°C for 30 min and fixed with methanol:acetic acid solution (3:1, Carnoy's solution). Drops of the cells in suspension were collected with impact on glass slides that were pretreated with Carnoy's solution (1 min) and washed with ice-cold water. Slides were subsequently air-dried and stained with 5% Giemsa solution for 2 min and rinsed in water, air-dried again and mounted with coverslips using Permount (Sigma-Aldrich). The spreads were imaged using a light microscope (Leica DM750 LED) and high-definition camera (Leica ICC50W), and chromosome numbers in individual spreads were counted.

### Reverse transcriptase polymerase chain reaction

Total RNA was extracted from BEFs infected with STEMCCA lentivirus (Day 2), and biPSCs cultured on iMEFs in GMTi medium at passages 2 and 10 by sequential purification using Trizol™ (Life Technologies) and an RNeasy Mini Kit (Qiagen). Reverse transcription (cDNA synthesis) was carried out using 2 µg total RNA with the Multiscribe™ reverse transcription kit (Life Technologies). Expression of the STEMCCA transgene was examined by performing polymerase chain reaction using primer pair 5′-TTCACATGTCCCAGCACTACC-3′ and 5′-GAAGCCGCTCCACATACAGT-3′ that specifically amplifies a 560 bp region of the cDNA synthesized from the polycistronic STEMCCA mRNA. Expression of the LT transgene was examined by performing polymerase chain reaction using primer pair 5′- GGCTACACTGTTTGTTGCCC-3′ and 5′- GCCTGCAGTGTTTTAGGCAC-3′ that specifically amplifies a 439 bp region of the cDNA synthesized from the LT mRNA.

### Transcriptome of bovine iPSCs

Colonies of biPSCs (passage 8) from three independent reprogramming events were selected for RNA-seq. Methods identical to that mentioned for blastocysts were used to extract mRNA, prepare libraries and sequence biPSC samples. After quality control and mapping, comparisons were performed to identify (1) differentially expressed genes between biPSCs and undifferentiated TSCs, and (2) differentially expressed genes between biPSCs and fibroblasts, or 16-CEs and fibroblasts, independently using R package EdgeR (Robinson et al., 2010). Transcriptome of primary bovine fibroblasts was from control samples in GEO GSE61027 (Green et al., 2015). Gene expression associated with bovine trophectoderm have previously been defined (Pillai et al., 2019a); genes associated with formation of the ectoderm, mesoderm and endoderm were as previously compiled (Maguire et al., 2013), and confirmed using gene ontology (GO) definitions (0007492, 0007398, 0007498). Complete biPSC RNA-seq datasets are available through NCBI GEO (GSE169624).

### Embryoid body formation

Feeder-free cultures of biPSCs were dissociated into single cells using TrypLE and resuspended in DMEM/F12 containing 10% FBS to achieve a concentration of 25,000 cells/ml. Rows of 20 µl hanging droplets for suspension culture of biPSCs were made on an up-turned lid (inner surface) of a 150 mm tissue culture dish. Inverting the droplets, the lid was placed on the dish that contained 10 ml PBS for maintaining humidity, and this setup was incubated at 37°C under an atmosphere of 5% CO_2_ for 2 days for embryoid body formation. Each embryoid body was then transferred to single wells of a low-attachment 96-well plate and cultured for 3 more days. Images were acquired using a stereo microscope (M80, Leica) and a high-definition camera (IC80HD, Leica).

### Teratoma assay

Feeder-free cultures of biPSCs were dissociated into single cells using TrypLE and resuspended in cold Matrigel^®^ diluted in DMEM/F12 (80 µg/ml final) at a concentration of ∼10^6^ cells in 200 µl. Cell suspension was loaded into a chilled 1 ml syringe with a 30G needle and transported on ice. Immunodeficient NSG mice [NOD.Cg-Prkdc^scid^Il2rg^tm1Wjl^/SzJ, Jax^®^ mice (Shultz et al., 2005)], 6-8 weeks of age, were subcutaneously injected at two to three sites in the flank and/or back, introducing 50-100 µl of cell suspension at each site. NSG mice were maintained under standard care and monitored for physical appearance of teratomas. At 6 weeks after injections, mice were euthanized and teratomas collected and fixed in 4% formaldehyde and held for histological processing. Paraffin embedding, cutting thin sections (4 µm thickness), and staining using Hematoxylin and Eosin were as previously described for mouse tissues (Morohaku et al., 2013, 2014; Tu et al., 2014). Morphological assessment for differentiation was performed by identifying the diversity of tissue types using methods in histopathology. Images were acquired using an upright light microscope (DM1000LED, Leica) and a high-definition camera (ICC50HD, Leica).

### Analysis of transcriptional regulation and pathways

Transcriptome of bovine 16-CEs were used as a reference to examine the equivalence of biPSCs for both authentication and advancing understanding of pluripotency regulation and pathways. Transcriptome of bovine 16-CEs was from GEO GSE52415 (Graf et al., 2014). The bovine fibroblast transcriptome was used as a normalizing dataset to delineate genes upregulated in pluripotency. After gene expression analysis for the 16-CE and biPSC datasets, transcription factor enrichment analysis was performed using ChEA3 (Keenan et al., 2019), to identify factors responsible for gene expression in 16-CEs and biPSCs. ReMap transcription-factor target gene set library was used to process transcriptomics data from these groups, yielding enrichment results/ranks. Distinct from this systems analysis, to precisely uncover the pluripotency-relevant transcriptional network, transcriptome of both 16-CE transcriptome and biPSC transcriptome were first compared to the transcriptome of primary bovine fibroblasts (Green et al., 2015) in order to reveal genes that are specifically upregulated in both. From this list, transcription factors were separated using a comprehensive reference list of 1595 compiled from three databases (de Souza et al., 2018; Weirauch et al., 2014; Zhang et al., 2012). Transcription factors that were commonly upregulated in both 16-CEs and biPSCs were examined for relative expression levels to identify highly expressed transcription factors, and their relevance to pluripotency was analyzed for functional association using the String database (Szklarczyk et al., 2017). Exploratory investigation and functional pathways associated with genes upregulated in biPSCs and 16-CEs compared to fibroblasts were analyzed using iDEP (Ge et al., 2018). Active receptor-mediated signaling in biPSCs was identified by integrated gene expression and protein interaction analysis using SPAGI (Kabir et al., 2018).

## Supplementary Material

Supplementary information
