# Supplementary material for: Efficient induction and sustenance of pluripotent stem cells from bovine somatic cells
Source: Biol Open. 2021 Nov 1;10(10):bio058756. doi: 10.1242/bio.058756 (PMC8565620; doi:10.1242/bio.058756)
Supplement: Supplementary information [file biolopen-10-058756-s1.pdf]

Table S1.

Click here to download Table S1

Biology Open • Supplementary information
